# Engineering Three-Dimensional Tumor Models to Study Glioma Cancer Stem Cells and Tumor Microenvironment

**DOI:** 10.3389/fncel.2020.558381

**Published:** 2020-10-16

**Authors:** Henry Ruiz-Garcia, Keila Alvarado-Estrada, Paula Schiapparelli, Alfredo Quinones-Hinojosa, Daniel M. Trifiletti

**Affiliations:** ^1^Department of Radiation Oncology, Mayo Clinic, Jacksonville, FL, United States; ^2^Department of Neurological Surgery, Mayo Clinic, Jacksonville, FL, United States

**Keywords:** glioma, tumor microenvironment, stem cell, bioprinting, organoids, organ-on-a-chip, tissue engineering, spheroids

## Abstract

Glioblastoma (GBM) is the most common and devastating primary brain tumor, leading to a uniform fatality after diagnosis. A major difficulty in eradicating GBM is the presence of microscopic residual infiltrating disease remaining after multimodality treatment. Glioma cancer stem cells (CSCs) have been pinpointed as the treatment-resistant tumor component that seeds ultimate tumor progression. Despite the key role of CSCs, the ideal preclinical model to study the genetic and epigenetic landmarks driving their malignant behavior while simulating an accurate interaction with the tumor microenvironment (TME) is still missing. The introduction of three-dimensional (3D) tumor platforms, such as organoids and 3D bioprinting, has allowed for a better representation of the pathophysiologic interactions between glioma CSCs and the TME. Thus, these technologies have enabled a more detailed study of glioma biology, tumor angiogenesis, treatment resistance, and even performing high-throughput screening assays of drug susceptibility. First, we will review the foundation of glioma biology and biomechanics of the TME, and then the most up-to-date insights about the applicability of these new tools in malignant glioma research.

## Introduction

Tumors are complex systems with dynamic and constant regulation of their different components during initiation, maintenance, and progression. Gliomas, and particularly glioblastomas (GBM), are some of the most comprehensively characterized cancers, and huge efforts have been done in an attempt to overcome the therapeutic plateau existing after current standard, and even experimental therapies. Unfortunately, despite all these efforts, there have not been significant advances in the way we treat our patients, and the cure is far from our current achievements.

Therefore, there is a need to reconceptualize the process in which GBM biology is being studied in order to find meaningful therapeutic approaches. In this setting, tumor microenvironment (TME) is an inevitable masterpiece to consider, as the inherent crosstalk between this and glioma CSCs is a defining driver of GBM heterogeneity, plasticity, and evolution.

Three-dimensional (3D) models, derived completely from patient tissue or incorporating biomaterials, are a new technology that has risen as a potential tool to better recapitulate TME dynamics. We aim to briefly summarize pertinent concepts about glioma biology and the biomechanics of the TME, and then describe recent advances and potential applications of this technology.

## Glioma Cancer Stem Cells

**Figure 1:**
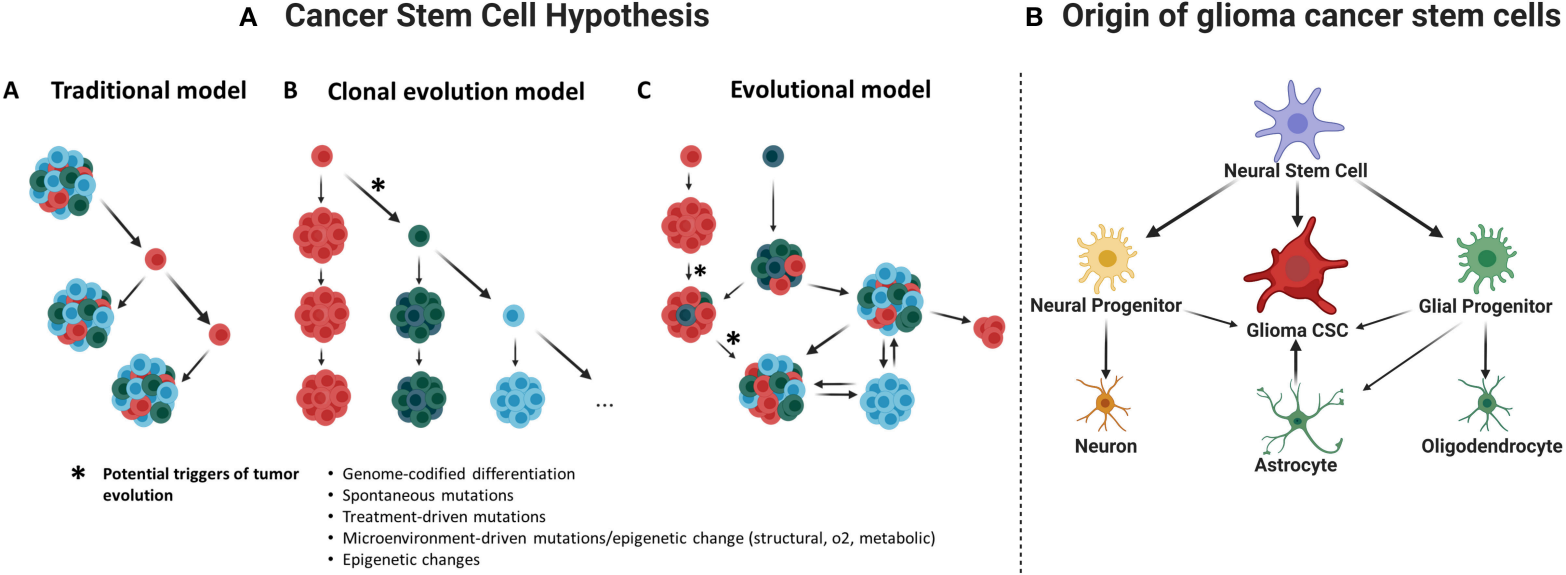
**(A) Cancer stem cell hypothesis**. This hypothesis suggests that a subset of cancer cells is responsible for tumor initiation and growth, having characteristics such as self-renewal and chemotherapy and radiotherapy resistance. (A) Traditional or hierarchical model. Suggesting the existence of a specific cancer cell population with stem-like properties that function as the tumor initiating cells, this population of CSCs would be sufficient to initiate and drive the tumor growth over time. (B) Clonal evolution model. It is proposed that many clones of CSCs would be functionally equivalent and able to maintain tumor growth; they would remain under constant genetic pressure that can introduce new characteristics and create new clones. (C) Evolutional or stochastic model. Random genetic or epigenetic events can transform any cell to a variety of cancer stem cells at any time within the tumor. This will presuppose a hierarchy of CSCs under constant evolution due to natural selection and genomic instability (Chen et al., 2010). **(B) Origin of glioma cancer stem cells**. Evidence suggests that neural stem cells, astrocytes, or oligodendrocyte precursor cells could be the origin of gliomas.

Glioma cancer stem cells (CSCs) were first described in early 2000s (Uchida et al., 2000; Hemmati et al., 2003; Singh et al., 2003; Galli et al., 2004) and were required to fulfill defining criteria of normal stem cells (Uchida et al., 2000; Hemmati et al., 2003; Singh et al., 2003; Galli et al., 2004). Therefore, glioma CSCs must be able to self-renew and grow tumors resembling its original histopathology. Several models have been suggested to explain CSC maintenance (Figure 1A); however, it is most probably that the evolutionary model of the CSCs hypothesis, or an even more holistic understanding, could better serve on this purpose (Chen et al., 2012).

Additionally, there is a lack of uniformity regarding the nomenclature of CSCs, which generates confusion and may redirect the research focus far from the study of CSC biology. While the term *stem cell* is used, this does not necessarily mean that CSCs derive from a distorted canonical stem cell (Figure 1B). Regardless of the true cellular origin of CSCs, the use of the term *stem cell* requires that these cells comply with at least functional defining criteria such ability to self-renew and generate different progeny with different hierarchies inside the tumor.

Several enrichment markers of stemness have been suggested to identify CSCs. BMI1, SOX2, NESTIN, OLIG2, NANOG, MYC, and IDI1 (inhibitor of differentiation protein 1), among others, are crucial transcription factors and/or structural proteins required for normal neural stem and progenitor cell (NSPC) function. These markers are shared between glioma CSCs and NSPCs. However, given that conventional methods used for CSC selection (CSC enrichment), such as flow cytometry, are limited in the use of intracellular proteins (as the ones stated above), several surface biomarkers like CD133, CD44, CD15, L1CAM, A2B5, and integrin α6 have been widely used instead. Interestingly, some of these surface biomarkers have been related to glioma cell–microenvironment interactions, which reflect the relationship between TME and glioma biology.

### Glioma CSC Markers and Its Interactions With the Tumor Microenvironment

#### CD133 (Prominin-1)

Human neural stem cells were identified for the first time by Uchida et al. (2000). The group harvested cells from fetal brain tissue and found that the isolated CD133^+^ population was able to fulfill the criteria required to be defined as stem cells. This finding prompted a scientific hunt for brain tumor stem cells, and soon after, CD133 was proposed as the first biomarker for glioma CSCs (Uchida et al., 2000; Hemmati et al., 2003). However, controversies about CD133 reliability raised after two independent groups showed that GBM CD133^−^ cells could also embrace stem cell properties such as self-renewal and differentiation *in vitro* and tumor formation *in vivo* (Beier et al., 2007; Joo et al., 2008; Wang et al., 2008; Wei et al., 2013). Furthermore, CD133^−^ population would tend to grow as adherent tumorspheres under conventional *in vitro* conditions and was proven able to give rise to cultures containing CD133^+^ glioma cells *in vitro* and *in vivo* (Wang et al., 2008; Chen et al., 2010). Overall, it was clear that glioma CSCs could also present as a CD133^−^ population.

CD133, also known as prominin 1, is a cell surface glycoprotein with five transmembrane domains. Given its superficial location, detection of CD133 may vary depending on several factors such as cell–microenvironment interactions and epigenetic influences. Careful analysis of its informational value is recommended as immediate cell–extracellular matrix (ECM) disassociation, extended *in vitro* culture, and/or equivocal epitope recognition may give rise to false-negative results (Clément et al., 2009; Osmond et al., 2010; Campos et al., 2011).

Although a definitive role for CD133 on glioma CSCs remains elusive, it is clear that the expression of CD133 may vary according to several interactions with the TME. For instance, changes in ECM composition (Logun et al., 2019) or decreased oxygen tension on the TME is related to higher CD133 expression (Platet et al., 2007; Soeda et al., 2009; Musah-Eroje and Watson, 2019) and faster expansion and retained undifferentiation in CD133^+^ gliomas cells. In the opposite direction, CD133 can lead to activation of PI3K/Akt signaling pathway leading to increased self-renewal and tumor formation (Wei et al., 2013), as well as interleukin 1β signaling-mediated downstream regulation of the TME through increased neutrophil recruitment (Lee et al., 2017).

#### CD44 (Hyaluronic Acid Receptor)

CD44 is a cell membrane glycoprotein that binds extracellular ligands present in the ECM, such as hyaluronic acid (HA) and osteopontin. These interactions promote cell motility toward ECM through the mechanotransduction involving CD44 linkage to cytoskeletal components (Tsukita et al., 1994).

As CD133^−^ glioma population was found to display stem cell–like properties, other markers of stemness were sought. The role of CD44 as a surface marker of glioma CSCs has been described by several authors (Tsukita et al., 1994; Anido et al., 2010; Xu et al., 2010); interestingly, CD44 would be the most common shared marked of stemness among CSCs derived from different malignancies (Mooney et al., 2016). CD44 has been associated with GBM aggressiveness through increased invasion and migration (upon binding with HA) (Radotra and McCormick, 1997; Brown et al., 2015), increased proliferation (Monaghan et al., 2000; Feng et al., 2014), and enhanced chemoresistance (Xu et al., 2010).

#### CD15 (SSEA-1)

CD15, also known as Lewis X or SSEA-1 (stage-specific embryonic antigen 1) is a cell surface carbohydrate antigen. CD15 was first suggested as a marker for glioma CSCs in 2009. Son et al. (2009) found that, in GBM, CD15^+^ cells possess a 100-fold tumorigenic potential when compared to CD15^−^ population. Furthermore, all CD15^+^ cells were also positive for CD133, whereas most of the CD133^+^ cells were CD15^+^ as well.

#### L1CAM

The neural cell adhesion/recognition L1 molecule (L1CAM or CD171) is a type 1 transmembrane glycoprotein of the immunoglobulin superfamily; this protein is normally found during central nervous system (CNS) development. In 2008, Bao et al. (2008) reported that L1CAM supported glioma CSC survival and clonogenicity in CD133^+^ cells through the regulation of Olig2 and the tumor suppressor p21. Furthermore, L1CAM function in GBM cell migration was determined by the same group; ADAM10 would cleavage L1CAM ectodomain, which then would activate EGFR and integrins (FAK-mediated process) to promote glioma CSC migration (Bao et al., 2008; Yang et al., 2011).

#### A2B5

A2B5 is a cell surface ganglioside present in glial precursor cells. Ogden et al. (2008) found that this epitope was also present in a sizable population of glioma-initiating cells; even more, most of the CD133^+^ cells were contained in the A2B5^+^ population. The authors were able to show that A2B5 renders stem cell properties even in CD133^−^ population (Ogden et al., 2008). Similar results were also presented by other authors (Tchoghandjian et al., 2010). Sun et al. (2015) showed that CD133^−^/A2B5^+^ population possesses great migratory and invasive potential and hypothesized that this could be characterizing the infiltrative cells of the invasive tumor front leading GBM posttreatment recurrence.

## Role of Tumor Microenvironment in Glioma Biology

TME is a crucial teamster of CSC heterogeneity, plasticity, and evolution (Charles et al., 2010). However, CSCs can reciprocally regulate the microenvironment. Glioma CSCs not only secure self-renewal (Man et al., 2014), malignant proliferation (Fan et al., 2010), and segregation into different tumor cells, but also interact in a multidirectional way with different tumor components such as the ECM, the cellular compartment (cancer-associated fibroblast, immune cells, differentiated neural cells, etc.), and even the blood–brain barrier (BBB) through tumor-derived pericytes in order to establish a favorable niche able to support further malignization and treatment resistance (Cheng et al., 2013). In this section, we will review this reciprocal crosstalk and its implications in glioma treatment behavior and resistance.

### Components of Glioma TME

#### Extracellular Matrix

The ECM constitutes the non-cellular compartment of the TME. This is a 3D molecular network built with water, proteins, and polysaccharides (Frantz et al., 2010). The specific composition of each ECM is driven by a real-time biochemical and biophysical feedback between cells and their surrounding microenvironment (Gattazzo et al., 2014). CSCs are in constant interaction with the ECM via several multifunctional transductors as we reviewed above (CD133, CD44, L1CAM, integrin α6, and others). CSCs are able to give rise to more differentiated cells that can later regulate the production of extracellular components in order to promote tumor niche homeostasis. It is noteworthy that abnormal ECM remodeling affects endothelial and immune cells, tumor angiogenesis, and drug penetration, thus influencing tumor aggressiveness and progression. Several ECM components, such as integrins, laminins, and cadherins, among others, have been linked to treatment response and patient survival (Figure 3) (Ljubimova et al., 2004; Lathia et al., 2012).

Laminins are a family of extracellular T-shaped heterotrimeric glycoproteins consisting of one **α**, one **β**, and one **γ** chain. In vertebrates, different genes codifying for five α, three β, and three γ chain exist. Although 45 combinations are possible, only 18 isoforms have been identified up to date (Laminin isoforms 8 and 9 are some of them). Laminins are usually located on basement membranes, a kind of “ECM residing in the outer layer of the blood vessels,” from where they can interact with other ECM molecules or cell receptors. Laminin location is usually α chain–specific, i.e., CNS tissue use to home α2 and α4 laminins (laminins containing an α2 or α4 chain, respectively). They can trigger downstream signaling for different biological processes including migration, adhesion, proliferation, and survival (Durbeej, 2010). In GBM, aberrant overexpression of α4 laminins has been described, and a positive correlation between their expression and tumor grade has been described (Sun et al., 2019). Particularly, laminin isoform 8 (α**4**β**1**γ**1**, or laminin **411** according to the new nomenclature) appeared overexpressed on GBM blood vessels and surrounding healthy tissue and was linked to higher recurrence and shorter survival (Ljubimova et al., 2001; Lathia et al., 2012). Furthermore, inhibition of laminin isoform 8 through CRISPR/Cas9 techniques has proven to suppress Notch pathway, rendering decreased intracranial tumor growth and longer survival in a glioma animal model (Sun et al., 2019). Lathia et al. (2012) showed that α2 laminins provided by perivascular non-CSCs and endothelial cells (ECs) were critical for GBM CSC maintenance and proliferation, promoting glioma CSC radioresistance through enhancing DNA repair. The use of laminin during routine *in vitro* culture of adherent glioma CSCs supports the importance of ECM proteins on glioma CSC biology. Overall, these observations highlight the role of the ECM on glioma treatment response.

Cadherins are surface glycoproteins involved in calcium-dependent cell–cell adhesion. The role of cadherins in glioma progression is not well-understood yet. However, the interaction between CSCs and other cellular components of the TME, such as those forming the white matter tracts that glioma cells used to migrate through or ECs from the BBB, has recently acquired great relevance (Drumm et al., 2019). Although previous studies reported differing results regarding the concentration level of cadherins and glioma cell invasion capacity, it was finally clear that rather than the concentration of cadherins available, the most important factor determining migration and invasiveness in GBM cell lines was the instability and disorganization of cadherin-mediated junctions (Barami et al., 2006). Cadherin E is common in epithelial cancers where, at some point along their evolution, cadherins undergo a process called *switching*. Despite its rarity within the CNS, cadherin E has been found in some GBM tumors. Here, contrary to epithelial tissues, high levels of cadherin E have been associated with aggressive invasiveness (Lewis-Tuffin et al., 2010). Cadherin 11 has been associated with increased migration and proliferation in different cancers. In GBM, cadherin 11 seems to support migration and survival *in vitro* and *in vivo*. Cadherin 11 also serves as a marker of mesenchymal phenotype, GBM subtype that is associated with worse prognosis (Kaur et al., 2012).

**Figure 2:**
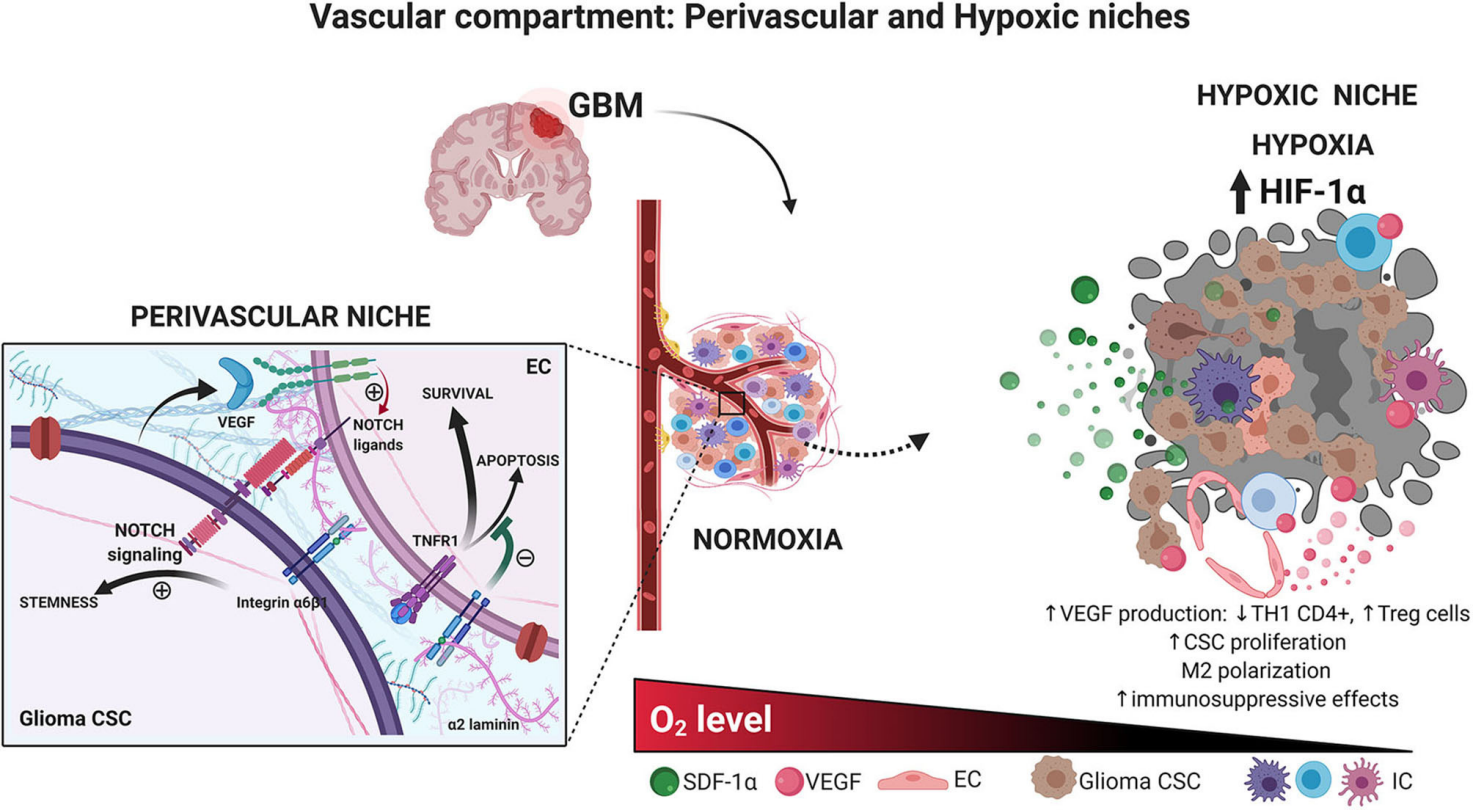
**Vascular compartment. Glioma CSCs and endothelial cells interaction in the perivascular niche**. Brain CSCs are located within a vascular niche interacting with the surrounded cells. The perivascular niche is critical to maintaining the CSC phenotype. The secretion of growth factor by endothelial, ECM, and hypoxic conditions preserves stem-like characteristic accelerating tumor growth. **Hypoxic glioma niche**. Decreased oxygen tension triggers the expression of HIF-related genes, which in turn increases the production of several factors such as VEGF, decreasing the T_H_1 CD4^+^ cells, and increasing T regulatory cells, macrophage polarization, immunosuppressive profile, and glioma CSC proliferation. Created with BioRender.com.

Integrins are heterodimeric transmembrane glycoproteins important in cell migration and cell adhesion. Although they are not a component of the ECM, they are key mediators of the interaction between different cellular components and the ECM. For this purpose, integrins function as receptors of laminins and fibronectins. In GBM, integrins are key in many complex processes, such as angiogenesis, tumor invasion, and proliferation (Nakada et al., 2013; Tilghman et al., 2016). The laminin-specific receptor, integrin α6β1, is highly expressed on perivascular glioma CSCs and is critical for their self-renewal and tumor formation capacity (Lathia et al., 2010). Integrin α6β1is also present in ECs from the perivascular glioma niche; upon laminin-binding, it has been shown to inhibit proapoptotic signals mediated by TNFR1, through NF-κB, by increasing cFLIP and XIAP, and promote EC growth (Figure 2) (Huang et al., 2012). Integrin α6 has also been reported as an enrichment CSC marker in GBM (Lathia et al., 2010). The invasive behavior of GBM CSCs seems to be mediated by another integrin. Integrin α3 was found overexpressed on glioma CSCs, especially in those leaving the tumor bulk and in those around the perivascular niche. Higher expression of integrin α3 correlated with increased migration and invasion via ERK1/2 signaling (Nakada et al., 2013). Integrin α5β1 is another integrin found in human GBM cells and was related to chemoresistance to temozolomide (Janouskova et al., 2012; Renner et al., 2016). Other integrins have been also reported to be involved in the crosstalk between CSCs and ECM (Haas et al., 2017).

#### Vascular Compartment: Perivascular and Hypoxic Niches

Similar to neural stem cells located in specific anatomical brain niches: the subventricular zone (SVZ) and the subgranular layer inside of dentate gyrus of the hippocampus (SGZ) (Quiñones-Hinojosa et al., 2006), glioma CSCs are present around the vascular compartment of the microenvironment. Researchers have suggested that this would represent a perivascular niche of glioma CSCs given the presence of CD133^+^ and NESTIN^+^ cells surrounding the tumor blood vessels (Calabrese et al., 2007). This statement was supported by the fact that culturing glioma CSCs along with ECs increases CSC proliferation *in vitro*, as well as by the deleterious effect of anti–vascular endothelial growth factor (VEGF) therapies on tumor growth (Calabrese et al., 2007). There is, in fact, an active crosstalk between the vascular endothelium and glioma CSCs. Bao et al. (2006) showed that glioma CSCs can secrete VEGF supporting the local angiogenesis. In turn, EC would produce Notch ligands that are widely known as a key determinant of CSC maintenance and proliferation and even would be able to recruit glioma CSCs and differentiate them into vascular pericytes (Figure 2) (Zhu et al., 2011; Cheng et al., 2013). As previously discussed, interactions between α2 laminins from the vascular basement membrane and the integrin α6β1 present in CSCs surface are important determinants of glioma CSC proliferation and migration. However, it is undeniable that CSCs are also present far from this hypothetical niche. Glioma CSCs from the hypoxic tumor core, as well as those moving away from the infiltrative border of the tumor bulk, are crucial for healthy tissue infiltration and tumor progression.

Hypoxic conditions have proven to facilitate glioma CSC self-renewal. Hypoxia-inducible factor 1α (HIF-1α) and HIF-2α as well as carbonic anhydrase IX, support CSC malignant potential and are correlated with poor patient prognosis (Li et al., 2009; Mohyeldin et al., 2010; Pistollato et al., 2010; Proescholdt et al., 2012; Xu et al., 2018). Thus, a hypoxic niche seems to be another realistic TME with a particular dynamic. HIF-1 would repress core-derived glioma cell differentiation through the suppression of Smad activation (Pistollato et al., 2009a,b), maintaining a higher number of stem cells that express greater levels of the DNA repair protein MGMT (O_6_-methylguanine-DNA-methyltransferase) and consequently turn to be more radioresistant (Pistollato et al., 2009a, 2010). Also, low oxygen tension leads vascular ECs within the hypoxic niche to produce several factors, such as VEGF-A, which confer a more aggressive behavior to glioma CSCs and polarize immune cells into an immunosuppressive phenotype, as it is demonstrated by tumor-associated macrophage (TAM) M2 polarization, increased regulatory T cells, and higher rates of PD-1^+^ CD8^+^ T cells, leading to treatment resistance to traditional and modern approaches such as immunotherapy (Escribese et al., 2012; Tamura et al., 2018, 2019a,b) (Figure 2).

#### Cellular Compartment

Apart from the ECM, glioma microenvironment contains a number of cell types as another component of the tumor dynamics. These cells actively interact with glioma cells and the ECM (Wang et al., 2018c); they can secrete several factors triggering different signaling pathways on CSCs, as well as modify ECM composition in response to bilateral tumor cell–non-tumor cell interactions.

Astrocytes play a key role in CNS homeostasis. Astrocytes present in GBM tumor as well as surrounding brain parenchyma are thought to modulate disease progression via displacement and degradation of astrocytic endfeet in the BBB (Watkins et al., 2014). As glioma cell migration has also been reported to happen along the vasculature, these cells could impact migration and invasion. Tumor-associated astrocytes have also been related to malignant transformation of surrounding healthy tissue, as well as resistance to chemoradiation (Chen et al., 2015, 2016; Wang et al., 2018c; Brandao et al., 2019).

Immune cells are an extremely important component of the TME. Myeloid linage cells, such as infiltrating peripheral macrophages and brain-resident microglia (tumor-associated macrophages/microglia or TAM), represent around 30 to 50% of the tumor mass (Lisi et al., 2017). From this group, around 40% are macrophages, 20% tumor-resident microglia, and the other 40% are myeloid-derived suppressor cells (Gabrusiewicz et al., 2016). Lymphocyte linage cells are also present in the TME. GBM-associated T cells and B cells (tumor-infiltrating lymphocytes or TILs) have been extensively described as having an exhausted phenotype; which correlates with their inability to control disease progression (Ma et al., 2018). This immunosuppressive tumor environment has been referred to as a “cold tumor.”

Brain tumor–associated mesenchymal stem cells have acquired relevance recently; these cells play a role in supporting glioma microenvironment (Behnan et al., 2014; Guo et al., 2014; Svensson et al., 2017; Yi et al., 2018). Shahar et al. (2017) showed that a high percentage of them in the tumor population have been correlated with poor clinical prognosis. Although fibroblasts are not a major component in GBM composition, GBM-associated stromal cells closely resemble tumor-associated fibroblasts found in other tumors. They are particularly located in the periphery of the tumor and have been found to enhance tumor growth (Clavreul et al., 2012).

### Biomechanics of ECM: Implications in Glioma Behavior

The effect of mechanical interaction on the cells was first proposed by His (1874); however, it was almost completely abandoned until recently. For many decades, research on cellular and molecular biology has been focused on intrinsic cellular biological processes without including biomechanical information about cell–ECM interaction (Paluch et al., 2015). In 1920, Alexander Forbes suggested that the collaborations between different scientific fields, such as biology and physics, will bring a better understanding of the living matter (Forbes, 1920). However, the communication flowed slowly until recent years in which new technologies facilitated access and cross-pollination of a huge amount of human knowledge. Recent multidisciplinary scientific work has led to important advances in understanding the influence of external physical forces in cell behavior, specifically mechanical forces such as tension, elasticity, stiffness, weight, friction, and others.

The process of sensing and responding to mechanical stimuli is known as mechanotransduction (Rice et al., 1973; Herberman, 1981; Wang et al., 1993). Cell migration, differentiation, proliferation, apoptosis, gene expression, and signal transduction (Sharma et al., 2019) are all influenced by mechanical stimuli (Chen and Wang, 2019; Moran et al., 2019).

Mechanical properties of the ECM can induce and maintain the stem-like phenotype in cancer cells. However, the response of cancer cells to the ECM mechanical properties varies between cancer types and even among cellular subpopulations within the tumors. For example, soft matrices induce the expression of self-renewal markers in melanoma CSCs, whereas stiff matrices induce their differentiation, but the opposite occurs in breast cancer (Nallanthighal et al., 2019). Moreover, the stiffness gradient in the TME in breast cancer is associated with specific CSC phenotypes, CSC CD24^−^/CD44^+^ localized in the tumor edges is quiescent, and CSC ALDH^+^ (more stem) is found in the tumor core (Sulaiman et al., 2018). Indeed, changes in the type and proportion of proteins that constitute ECM can alter its stiffness by modifying the cross-linking ratio, amount of specific proteins, and cell–ECM interactions. Thus, it has been shown that these changes may induce FAK, FGF5, and JKT signaling activation, which contributes to the CSC phenotype (Cazet et al., 2018). Furthermore, mechanical properties in the ECM composition and organization could induce epithelial-to-mesenchymal transition (EMT) in cancer cells, this phenotype confers stem cell–like properties to cancer cells and is associated with chemoresistance and relapse (Singh and Settleman, 2010).

#### Biophysical Properties of Glioma ECM

The characterization of the mechanical properties of soft tissues in humans represents a great challenge because these are integrated by numerous components. Individual analysis of each of these components has granted insight into tumor mechanobiology; however, under real conditions, these elements work coordinately supporting tumor progression. A roadblock to overcome in order to better understand the TME mechanobiology is that tumor mechanical data obtained from biopsies may not be completely representative, as their value is relative to the location from where the samples were taken.

Young's modulus [force/area, in N/m^2^ or Pascals (Pa)] refers to the amount of force needed to deform a substance, and it is commonly used to measure tissue stiffness. Brain tumors have mechanical properties different from those of their surrounding tissue (Chauvet et al., 2016; Pepin et al., 2018). On average, normal brain stiffness is lower than 200 Pa; however, stiffness in gliomas gradually increases accordingly with glioma aggressiveness (World health Organization grade) and ranges from 100 to 10,000 Pa (Miroshnikova et al., 2016). These differences in tumor stiffens have been explained by elevated levels of collagen IV and HA, which turn to be associated with tumor progression.

Lately, a key piece of mechanotransduction has been described. For instance, Chen et al. (2018) described the role of the mechanosensitive ion channel PIEZO1 in glioma. PIEZO1 is a transmembrane protein that locates at various tumor cell regions including focal adhesions. Physical force–induced membrane tension opens the channel to allow ion permeation, leading to a genetic interaction with integrin FAK signaling, which in turn increases ECM proteins production (laminins, HA synthases, etc.) and glioma aggressiveness. Overall, this leads to an increase in tissue stiffening, which further promotes PIEZO1 upregulation in a reciprocal manner promoting glioma invasion and proliferation (Chen et al., 2018).

Other authors have also published results in accordance with the positive relationship between ECM stiffness and glioma aggressiveness. Thus, glioma cells with aberrant expression of EGFR have been shown preference for stiffer microenvironments (Sivakumar et al., 2017), and softness of the glioma tissue positively correlated with higher tumor grade and IDH1 mutation (Pepin et al., 2018). Overall, these findings are a call for more comprehensive studies on ECM–CSC interactions.

#### Components of ECM and Glioma Aggressiveness

The soft physical consistency of the brain tissue is owed to its ECM composition, which is abundant in proteoglycans such as hyaluronan, tenascin C, brevican, neurocan, and phosphocan (Miroshnikova et al., 2016). CSCs in the brain are exposed to this exclusive microenvironment in which the matrix–cell interaction activates pathways for stem phenotype maintenance, ECM remodeling, and proliferation (Manini et al., 2018). For instance, CD44 is highly expressed in gliomas; this protein interacts with HA to enhance CSC properties by activating NANOG (Pietras et al., 2014; Wang et al., 2017). Integrins are cell-surface proteins that work as transmembrane links between ECM and intracellular cytoskeleton by bidirectional signaling. In cancer, the expression and localization of integrins vary from normal cells; for instance, in GBM, integrin α6 (a receptor for the ECM protein laminin) is overexpressed; the interactions between integrin α6 and laminin regulate CSC distributions and maintenance, as we have mentioned above (Lathia et al., 2010). Integrin α3 is also highly expressed in glioma CSCs, this integrin interacts with fibronectin and laminin and has been localized in the CSC niche, promoting glioma invasion via ERK pathway (Nakada et al., 2013). Integrin α7 is aberrantly expressed in aggressive gliomas and correlates with poor prognosis, is highly expressed especially in glioma CSC subpopulations, and promotes tumor growth and spreading via AKT (Haas et al., 2017).

#### ECM and Glioma Treatment Response

Heterogeneity and genetic plasticity present in GBM allow for numerous mechanisms of therapeutic resistance. Interaction between glioma cells and the ECM plays a fundamental role as drivers of these two GBM properties. The ECM can induce EMT in CSCs, which confers stem-like properties as well as chemoresistance and radioresistance. Fibulin-3 is an ECM protein absent in normal brain tissue but upregulated in gliomas; this protein activates Notch signaling to promote resistance to apoptosis, chemoresistance, and tumor growth (Hu et al., 2012). Wtn proteins from the ECM confer high chemoresistance and radioresistance to temozolomide (Auger et al., 2006; Han et al., 2017). Additionally, the overexpression of fibrillary proteins in the glioma ECM has been reported as physical barriers against drug dissemination (Shergalis et al., 2018).

### Modeling TME to Study Treatment Resistance

As we have previously described, TME characteristics, such as ECM composition and biomechanical properties, as well as its vascular and cellular compartments, clearly influence CSC behavior and treatment response. Thus, it is not surprising that the use of two-dimensional (2D) cell cultures is associated with poor representation of the therapeutic response to chemotherapy and radiotherapy when compared to original tissues or even 3D models (Storch et al., 2010; Luca et al., 2013).

#### Studies in Radiobiology and Radioresistance

Bauman et al. (1999) pioneered the studies on radiation responses in 3D glioma models. In 1999, the authors used glioma tumor spheroids implanted into a gel matrix of collagen type I to study the effect of radiation on proliferation (Ki67), apoptosis, and invasion. After applying single and fractionated doses of (Pistollato et al., 2010). Co irradiation delivered at 200 cGy/min, they found differences in these variables according to the regional distribution along the spheroid. Cells at the surface of the neurosphere were more affected by radiation, whereas apoptosis and proliferation decrease was minimal or null at the core of the neurosphere. The invasion was affected in a dose-dependent manner, whereas fractionation seemed to confer associated with partial recovery. Taken together, this model showed to resemble qualities of *in vivo* models of malignant gliomas. Despite these results, efforts were not resumed until 15 years later (Jiguet Jiglaire et al., 2014; Yahyanejad et al., 2015) reported on the simultaneous comparison of a 3D spheroid model and an *in vivo* rodent model with regard to response to radiation therapy. They used the small animal radiation therapy platform (X-RAD SmART®) and performed a delivery plan delineating the tumor as gross total volume and the brain as an organ at risk (OAR), planning target volume was equal to GTR (225 kVp at 12 mA, 300 cGy/min). They found that the 3D model could be reliable for radiation efficacy evaluation (Yahyanejad et al., 2015). In this same line, 3D glioma models have been also proven effective in studying glioma radiosensitivity to different types of radiation modalities. Chiblak et al. (2016) used 3D clonogenic survival assays on patient-derived neurospheres and the classical radioresistant U87-MG GBM cell line to study radiosensitivity and measure the relative biological effect (RBE) of photon, proton, and carbon irradiation. The authors found that carbon irradiation RBE ranged from 2.21 up to 3.13 when compared to photon radiation and that the inability to repair double-strain DNA breakdowns after heavy ion irradiation could be a potential explanation for their findings (Chiblak et al., 2016).

In an attempt to represent not only the cell–cell interactions of the TME but also cell–ECM interactions, Jiguet Jiglaire et al. (2014) studied the role of 3D scaffolds based on an HA-rich hydrogel in the screening of radiation and chemotherapy response of commercial or patient-derived glioma cell lines. The 3D model showed good morphological representation when compared to patient-derived tissue specimens. Commercial cell line U87-MG did not show differences in radiation response when 2D and 3D cultures were compared; however, patient-derived glioma cell lines were proven radioresistant when cultured on the 3D model but not in conventional 2D cultures (Jiguet Jiglaire et al., 2014). Interestingly, the authors did not account for the difference in HA concentration between 2D and 3D cultures. Gomez-Roman et al. (2017) showed that, under the same culture conditions, architectural modifications (2D vs. 3D) did not generate differences in radiotherapy resistance. Conventional 2D cultures were compared with 3D cultures using scaffolds of polystyrene, both coated with laminin and using regular serum-free stem cell media. 3D cultures improved the morphological representation including hypoxic gradients characteristic of TME, but this did not represent an increment in radioresistance. When the 3D culture was enriched with additional laminin, increased radioresistance was evident (Gomez-Roman et al., 2017).

Overall, it is clear that *in vitro* models need to be perfected in order to better represent glioma biology and treatment response; a complex representation of TME biomechanical factors, ECM, and cellular compartments is necessary in order to achieve that goal. Foundations, adequate nomenclature, and applications of traditional and novel 3D models in glioma CSC research will be described in the next section.

## Three-Dimensional Models in Cancer Research

Every cell in the human body is immersed in a three dimensional microenvironment that regulates its behavior and potentially, its fate. In this setting, *in vitro* models aiming to understand glioma biology in order to develop effective therapies should ideally mimic the TME. Unfortunately, the traditional methods used for this purpose usually include the use of 2D cell lines cultures, which lack the aforementioned ideal requirement. The 2D approach introduces inherent limitations such as (1) genetic and epigenetic modifications due to the lack of CSC-TME interactions (Figure 2) (De Witt Hamer et al., 2008; Luca et al., 2013; Wang et al., 2018c), (2) absence of O_2_, nutrients and pH microenvironment gradients (Figure 2) (Bristow and Hill, 2008; Mikhailova et al., 2018), (3) lack of physiological inputs from other metabolically active organs such as liver, kidney, etc., and (4) genomic alterations after long-term culture (De Witt Hamer et al., 2008; Torsvik et al., 2014). Additionally, after a successful initial experimental phase involving 2D cultures, the next conventional step is usually carried out through animal studies, which are expensive and time-consuming. Furthermore, animal models have also demonstrated limited chances to translate these data into human outcomes (Shafiee and Atala, 2016).

To overcome these limitations, a great variety of 3D models or biocomplexes incorporating biomaterials and different tumor cells have been studied (Chang et al., 2010; Shafiee and Atala, 2016). Biomaterials are synthetic or natural nontoxic elements that can be engineered to obtain specific physicochemical characteristics; this attribute makes them a perfect fit to create biomimetic platforms able to resemble the 3D TME (Hildebrand Hartmut, 2013). Current technologies allow for recreating controlled patterns and stiffness properties of the ECM, which might provide the required microenvironment for CSCs to mimic their *in vivo* behavior (Shafiee and Atala, 2016). *In vitro* 3D models in cancer research can be classified in spherical cancer models (which include the tumorspheres or neurospheres), organoids, and 3D scaffolds.

### Spherical Cancer Models

Spherical cancer models consist of sphere-like structures mainly or totally composed of cancer cells (Friedrich et al., 2009). Due to their easy production, they are the most commonly used 3D *in vitro* model. There are several spherical cancer models described since almost four decades ago; however, their use and nomenclature have been confusing ever since. For instance, the terms *sphere* or *spheroid* have been misused in the literature to refer to cellular aggregates. Although both are a specific type of spherical cancer models, this misuse should be avoided as cellular aggregates differ from spheroids and spheres. Contrary to spheroids, aggregates are not compact enough to allow for manipulation and transfer; they easily detach and have no spherical geometry and probably no cell–cell and cell–matrix interactions, which impact their biological features (Weiswald et al., 2015).

**Figure 3:**
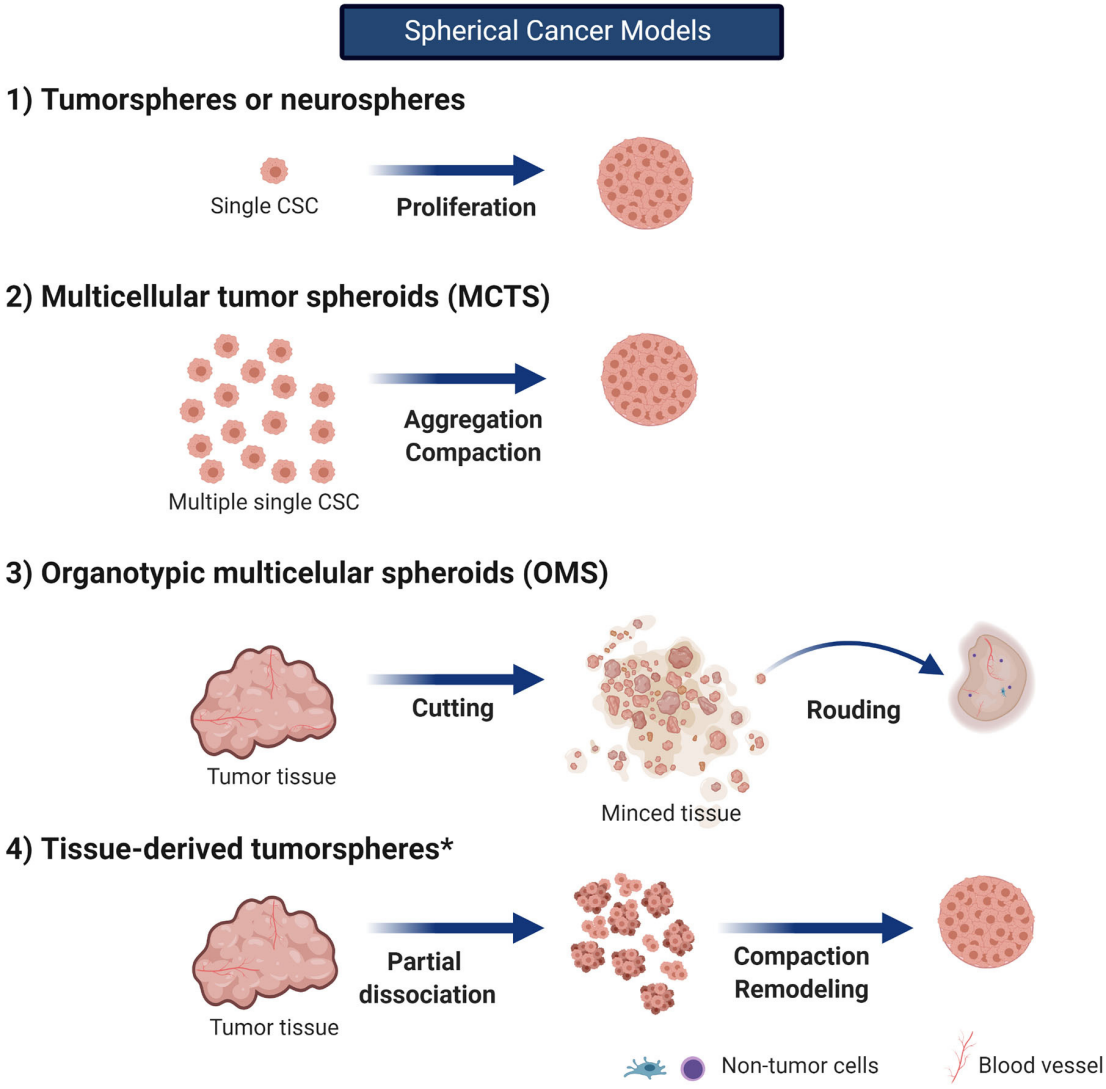
**Spherical cancer models**. *in vitro* 3D models in cancer research can be classified in spherical cancer models, organoids, and 3D scaffolds (3D and 4D bioprinting). Spherical cancer models are commonly use models. Created with BioRender.com. *no described for gliomas.

Weiswald et al. (2015) classified the spherical cancer models into four principal types (main features and culture conditions are described in Table 1 and Figure 3, with an emphasis on glioma research). Tumorspheres, or neurospheres in the case of gliomas, are one of the four different types of spherical cancer models. They are proliferations of single-cell suspension of tissue-derived cancer cells, circulating cancer cells, or established cell lines (clonal expansion) and were first described for gliomas by Singh et al. (2003). Tumorspheres are able to maintain CSC multipotency, resemble 3D interactions, and even resemble the tumor gradient of oxygen and nutrients. Thus, they present a quiescent necrotic core and a more proliferative outer layer. Tumor spheroids can be grown in suspension in the regular specific stem cell media or submerged in a gel, which has allowed them to be used as an important tool for high-throughput drug screening (Mirab et al., 2019).

**Table 1:** Different types of spherical cancer models in cancer biology research.

**Spherical cancer model**	**Origin**	**Culture conditions**
**Tumorspheres** Other names: • Tumor spheres • Neurospheres	Proliferations of single-cell suspension of tissue-derived cancer cells, circulating cancer cells or stablished cell lines (***clonal expansion***) (Singh et al., 2003) No non-neoplastic cells are present *First described for gliomas by Singh et al. (2003)	Serum-free medium (no FBS) FGF-2 and EGF are required (stem cell medium) (Lee et al., 2006; Claes et al., 2008) Grown in low-attachment conditions (i.e., no laminin-coated plates in case of glioma CSCs) Low seeding density to avoid aggregation and to foster ***clonal expansion***
**Multicellular tumor spheroids (MCTS)** Other names: • Tumor spheroids	Aggregation and compaction of single-cell suspension from well-stablished cancer cell lines Rarely from single-cell suspension of tissue-derived cancer cells Heterotypic MCTS including CSCs and noncancerous cells have been reported (co-cultures) *First escribed for gliomas by Mashiyama et al. (1989)	Serum-supplemented medium (FBS or FCS) No additional growth factors Grown in non-adherent conditions promoting aggregation of cells Two culture methods: liquid overlay (LOC) and spinner cultures (SPC) (Watanabe et al., 1999). Usually by several weeks The use of U87 cells was described by Bell et al. MCTS size ranges from 400 um to 1000 um after aggregation and compaction (Bauman et al., 1999; Bell et al., 1999, 2001)
**Organotypic multicellular spheroids (OMS)** Other names: • Organotypic spheroids	Rounding of non-dissociated *ex vivo* fragments directly from surgical specimens (0.3–0.5 mm for glioma tissues) (De Witt Hamer et al., 2008) Maintain stromal components (macrophages and tumor vessels)	Cultured with liquid overlay method until they round up (2 to 5 days) Tumor microenvironment has been shown to be present up to 70 days of culture Improved genomic stability when compared to well-stablished and primary glioma cell lines Cryopreservation of glioma OMS is well-tolerated
**Tissue-derived tumor spheres**	Remodeling and compaction of partially dissociated (mechanically or enzymatically) tumor tissue No non-neoplastic cells reported inside the sphere	FBS-supplemented or stem cell medium

*FBS, fetal bovine serum; FCS, fetal calf serum; FGF, fibroblast growth factor; EGF, epidermal growth factor; CSC, cancer stem cell; ECM, extracellular matrix; MCTS, multicellular tumor spheroid; OMS, organotypic multicellular spheroids. Based on the classification of Weiswald et al. (2015)*.

In the gel-embedded systems, cancer cells are surrounded by an artificial matrix to simulate cell–ECM interaction. In this strategy, the biomaterial properties can be modified to imitate ECM mechanical and structural characteristics, which could help resemble the TME. Currently, several commercial matrices such as Matrigel® are commercially available for this purpose. Agarose hydrogels conformed as microwells allowed the size control of tumoroids to evaluate the effect of therapeutic drugs, this technology can contribute to the advancement of personalized medicine (Mirab et al., 2019).

To date, neurospheres are the most common type of tumor spheroid used in glioma research (Table 2). Patient-derived neurospheres are grown in enriched EGF/bFGF media under low attachment conditions; when these factors are replaced by serum, glioma CSCs phenotypically change their appearance, loosen cellular adhesions, and turn the neurosphere into a 2D cell culture with decreasing CSC marker expression and telomerase activity (Lee et al., 2006; Claes et al., 2008). Furthermore, it has been reported that neurosphere-derived cells retain their ability to grow diffusely infiltrating tumors, whereas the same glioma cells grown under serum conditions could only produce well-demarcated tumors (Lee et al., 2006; Claes et al., 2008).

**Table 2:** Current glioma research using 3D platforms.

**3D model**	**Features**	**Developments and applications**
**Spheroids**	Mirror glioma CSC multipotency Maintain tumor cellular heterogeneity 3D cell-to-cell interactions Biomimetic 3D distribution* • Necrotic core • Inner quiescent layer • Outer proliferative layer Artificial low-adhesion cell growth microenvironment (Velcro type) Inability to organize in tissue-like structures (in case of tumorspheres) Cost-effective/highly reproducible	Drug screening using microfluidics-based chips
**Organoids**	Created with organoid technology Mini brains resemble non-tumor environment Tumor initiation in mini brains can be obtained by • Genome edition • Glioma CSC transplantation Glioma organoids can derive from pure tumor tissue	Study of gliomagenesis by introducing oncogenic mutations by gene editing strategies in brain organoids Study of tumor progression and Invasion Study of angiogenesis Study of tumor non-tumor interactions (in mini brains developing tumors) Biobank and drug screening for personalized medicine
**Scaffolds** *Hydrogels*	3D biocompatible polymeric matrices Structured microarchitecture (pores, groves, channels, etc.) Can introduce ECM proteins: HA, etc. Stiffness regulation Biodegradable Smart materials	Study of glioma CSC-vascular niche interactions Study of mechanisms underlying glioma migration Study of the role of ECM stiffness on glioma behavior (simulating diseased and healthy brain tissue) Study of cell-cell interaction
**Organ-on a -chip**	3D biomimetic system Continue and digitally controlled flow Flow ranges from *m*L to *p*L Tracks and regulates different conditions Interconnects multiple microenvironments	Study of glioma CSC-vascular niche interactions Drug screening using microfluidics-based chips Study of response to magnetic thermal therapy

3D, there-dimensional, mL, microliter; pL, picoliter; ECM, extracellular matrix; CSC, cancer stem cell; *under certain conditions.

Multicellular tumor spheroids (MCTSs, usually known as glioma tumor spheroids) are the second type of spherical cancer model. The initial development of MCTSs was based on the work of Sutherland et al. (1971) dating back to the early 1970s, and its role in glioma research appeared in 1989 with Mashiyama et al. (1989) Despite the model was described a long time ago, it is still a valuable tool to consider for high-throughput screening of several treatments such as radiation, drugs, and nanotherapeutics (Bauman et al., 1999; Yahyanejad et al., 2015; Oraiopoulou et al., 2017; He et al., 2020). Different culture methods and techniques have been developed (Watanabe et al., 1999); but all of them involve seeding an elevated number of cells under non-adherent conditions and promoting their aggregation and compaction. Usually, commercial cell lines are cultured with medium supplemented with serum (such as with U87 or T98G cells) (Bell et al., 1999, 2001; Oraiopoulou et al., 2019), but the use of patient-derived cell lines has also been described in several cancers (Weiswald et al., 2015; Yahyanejad et al., 2015). Even when a main mechanism of spheroid formation is aggregation, these spheroids are not simple cell aggregates; they form a very tightly packaged structure with intermediate junction between adjacent cells, and—as in any spherical cancer model—a differential dynamics is established from the core to the peripheral layer of cells (Bell et al., 1999, 2001).

Organotypic multicellular spheroids (OMSs, also known as organotypic spheroids) are the third type of spherical cancer model. These are rounded, non-dissociated *ex vivo* fragments of tumors obtained directly from surgical specimens. They maintain the non-tumor components such as immune cells and ECM for up to 70 days of culture and have demonstrated more representative GBM genetic profile when compared to primary cell cultures even after several weeks of culture. Although cryopreservation of glioma OMSs has been proven well-tolerated, the limited availability of GBM tissue is a highly restraining factor to introduce this model into regular glioma research (De Witt Hamer et al., 2008).

The last spherical cancer model described by Weiswald et al. (2015) refers to the tissue-derived tumorspheres; however, they have been not described for glioma. All the above mentioned models are described in Table 1 and Figure 3.

### Organoids

Organoids are self-organizing, 3D microscopic structures that are derived from individual stem cells growing in an *in vitro* environment. They can recapitulate histoarchitecture and cellular composition, as well as physiological aspects of the mature primary tissue they are derived from Eiraku et al. (2008), Muguruma and Sasai (2012), Lancaster et al. (2013), and Lancaster and Knoblich (2014). In general, organoids can be obtained from adult stem cells (ASCs) or pluripotent stem cells (PSCs) (Tuveson and Clevers, 2019). Although no neural tissue can be obtained from ASCs such as neural stem cells to date, PSC technologies have allowed for the creation of brain organoids from induced PSCs (iPSCs) (Eiraku et al., 2008; Muguruma and Sasai, 2012; Lancaster et al., 2013; Lancaster and Knoblich, 2014). The landmark article published by Lancaster et al. (2013) opened the door to different avenues in developmental and cancer research. The group created brain organoids, also known as cerebral organoids or mini-brains, presenting various discrete but interdependent brain regions. Complying with the definition of organoid, these brain organoids showed a cerebral cortex containing progenitor cells that self-organize and develop into different mature cortical neuron subtypes, as well as a primitive ventricular system and choroid plexus.

**Figure 4:**
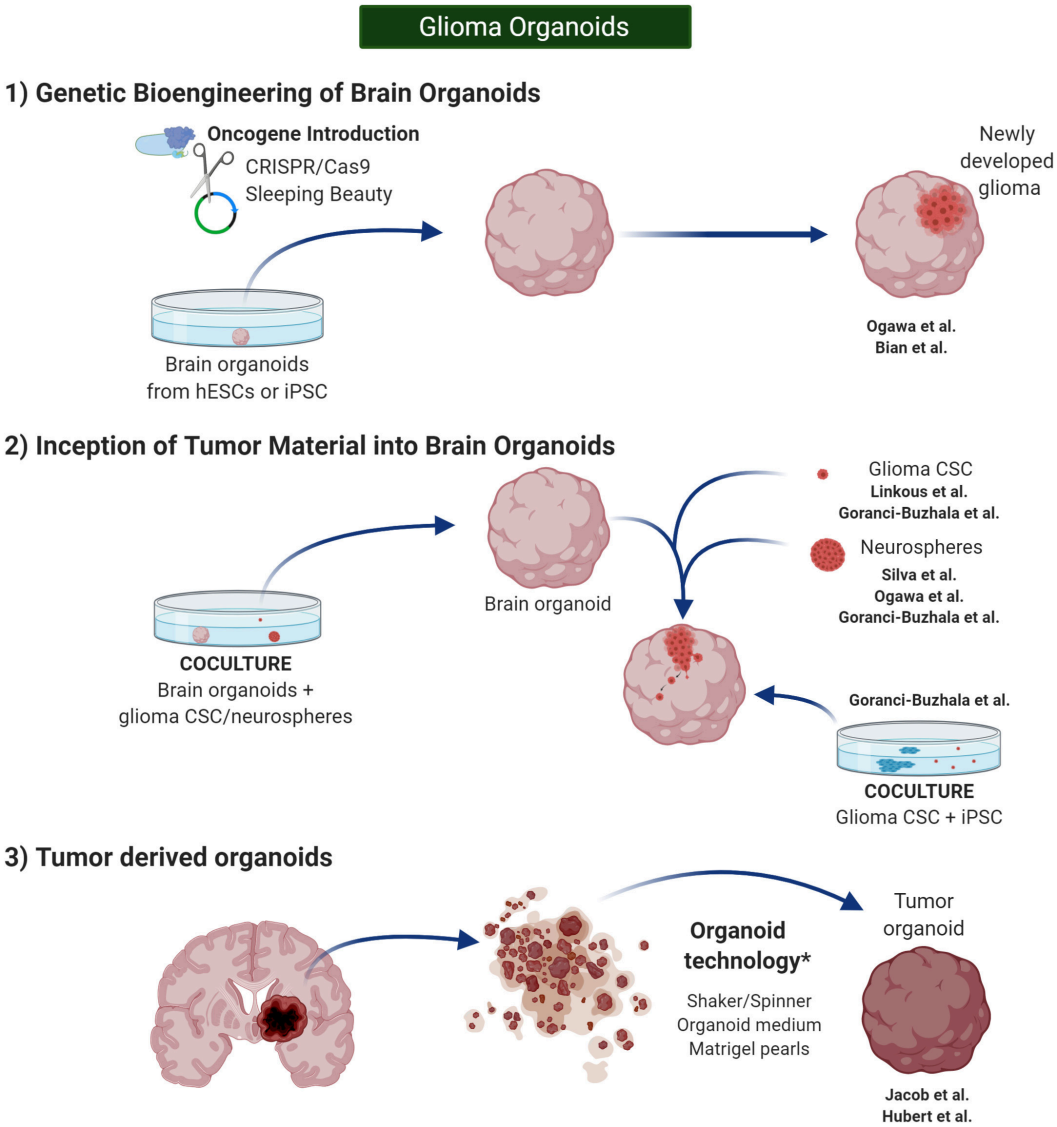
**Glioma Organoids**. *in vitro* 3D models in cancer research can be classified in spherical cancer models, organoids, and 3D scaffolds (3D and 4D bioprinting). Glioma organoids are produced using protocols similar to the one published by Lancaster et al. (2013) and Lancaster and Knoblich (2014). They need to be cultured under shaking conditions to increase diffusion of the nutrients. Created with BioRender.com.

Furthermore, despite that developmental biology defines the term “organoid” in this very pure manner, alternative protocols have been described in glioma research, and the term has been adapted to introduce glioma CSCs or even entire pieces of glioma tissue, containing a wide variety of glioma cells, as the origin of tumor organoids. Thus, glioma CSCs or tissue will be treated using organoid technologies in such a way that the newly developed “organoid” will recapitulate the glioma TME rather than a normal brain histoarchitecture. Overall, these different approaches have led to three groups of organoids (Figure 4).

#### Glioma Organoids: From Genetic Bioengineering of Brain Organoids

Genome engineering has been used to generate glioma tumor models in PSC-derived brain organoids or “mini-brains.” CRISPR/Cas9 mutagenesis and Sleeping Beauty (SB) transposon-mediated gene insertion have served for this purpose by introducing clinically relevant oncogenic mutations into healthy human cerebral organoids in order to develop glioma tumors (Bian et al., 2018; Ogawa et al., 2018) (Figure 4–1).

Ogawa et al. (2018) used human cerebral organoids cultured as described by Lancaster and Knoblich (2014) thus, organoids were grown and matured for 4 months. It was at this point, when the organoids already presented normal cortical structures and markers, that CRISPR/Cas 9 technology was used to mediate homologous recombination of the oncogene HRas^G12V^ into the TP53 tumor suppressor locus. This genomic insertion would simultaneously represent the disruption and truncation of the tumor suppressor gene TP53, as well as the introduction of the oncogene HRas^G12V^, which codes for the expression of RAS protein. Two weeks after this process, transduced cells can be initially observed through tdT and GFP signals, and by 8 weeks, almost 6% of the cells in the organoids are cancer cells. Therefore, this methodology allows for direct observation of tumor initiation, as well as continuous microscopic observations of tumor development. Consistent with other authors, the genetic alteration introduced by Ogawa et al. (2018) led the glioma organoids to show a molecular signature proper of gliomas of the mesenchymal subtype (Friedmann-Morvinski et al., 2012).

The group of Jürgen Knoblich, who initially published the landmark paper on cerebral organoids together with Lancaster, presented a similar approach. Human cerebral organoids were developed from human embryonic stem cells or iPSCs as previously described (Lancaster et al., 2013; Lancaster and Knoblich, 2014). By the end of the neural induction period, around day 11, SB transposon–mediated gene insertion for oncogene amplification and CRISPR–Cas9 technology for tumor-suppressor gene mutation were used to introduce 18 different single mutations or amplifications, and 15 of their most clinically relevant combinations in neuro-oncology. One of the newly developed clusters of organoids (containing three different combinations of genetic aberrations: GBM-1, GBM-2, and GBM-3) presented a glioma signature with particular upregulation of GBM-related genes and phenotype. These organoids proved to be viable and able to expand after heterotopic renal subcapsular engrafting (Bian et al., 2018).

#### Glioma Organoids: From Inception of Tumor Material Into Brain Organoids

In a similar manner, the development of glioma tumors has been also proven in healthy brain organoids after coculture with GBM CSCs or tumorspheres (da Silva et al., 2018; Ogawa et al., 2018; Linkous et al., 2019) (Figure 4–2).

Linkous et al. (2019) developed a cerebral organoid model of glioma called GLICO; they showed that glioma CSCs were able to infiltrate healthy cerebral organoids of different ages by coculturing them for 24 h. Considerable tumor growth was found 1 week after coculture, and the resulting tumors resemble original patient tumors genetically, functionally, and morphologically when examined 2 weeks after CSC inception. Apart from the already expected fact that the organoids represent radioresistance and chemoresistance of the primary tumor in a better way than 2D cultures, it was interesting that the non-cancerous microenvironment of the organoids seemed to support the maintenance, viability, and growth of the glioma CSCs (Linkous et al., 2019). Similarly, da Silva et al. (2018) reported the use of early-stage 12-day mouse ESC-derived brain organoids and GBM spheres in order to develop glioma organoids. Coculture of these two elements for 48 h resulted in a 100% rate of spontaneous infiltration of GBM cells into the organoids. The final size ranged from 300 to 800 μm, which would make them pertinent for medium- or high-throughput screening applications (da Silva et al., 2018).

As part of the same effort described in the previous section, Ogawa et al. (2018) studied the oncogenic potential of the glioma cells they had created in human brain organoids by CRISPR/CAS9-assisted mutagenesis, as well as patient-derived glioma CSCs. Similar to da Silva, the authors cocultured spheroids of either of these two types of cells with intact human mature brain organoids. Spheroids from tumor cells created by mutagenesis were able to invade the organoids and represent 30% of them by day 24. Spheroids from patient-derived cells presented different invasion capacity. In general, results from these two approaches correlated with results from *in vivo* experiments using immunocompromised mice (Ogawa et al., 2018).

Recently, Goranci-Buzhala et al. (2020) described three different methods to engineer the interaction between glioma and brain organoids. The authors compared two strategies similar to those described before by Linkous, Ogawa, and da Silva (glioma CSCs + brain organoids as well as glioma neurospheres + brain organoids) and an additional assay able to engineer this interaction as well (by coculturing glioma CSCs and iPSCs to develop organoids under conditions similar to those described by Lancaster et al., 2013; Lancaster and Knoblich, 2014). Thus, by using conventional and novel imaging technologies, the authors showed that the three models allowed analyzing glioma CSC invasion and patterns of invasion when primary and recurrent gliomas were compared. However, they also found that the latter model may not be the most suitable and that glioma CSCs tend to present enhanced tropism for mature brain organoids (Goranci-Buzhala et al., 2020).

Overall, the development of glioma tumors on cerebral organoids, either by mutagenesis or glioma inception, has opened a door to study brain tumor initiation, progression, and treatment. The presence of tumor and non-tumor microenvironments together at the same time allows for the study of the interactions between these two important components. Furthermore, the nature of these two approaches diminishes the need for patient-derived tumor tissue and animal xenotransplantation models to test patient-specific drug responses (Gao et al., 2014; Tuveson and Clevers, 2019).

#### Glioma Organoids: From Tumor Material Alone

Similar to the advances in other cancers' research, efforts in glioma research have aimed to accurately recapitulate the TME as much as possible. The previous models of organoids offer the possibility to study normal tissue–tumor interactions; however, key elements of the cellular components of the glioma tumor are missing given that only neural and glioma cells are available.

Jacob et al. (2020) used organoid technology to develop glioma organoids derived from tumor tissue, able to preserve cytoarchitecture and maintain different cell–cell interactions (Figure 4-3). They cultured the tissue in organoid medium and put it on an orbital shaker in order to increase nutrient and oxygen diffusion. Thus, by the end of the second week, a rounded organoid was appreciated, many of these organoids were able to retain their CD31^+^ vasculature, and resemble hypoxic niches 300 μm far from these vessels. Robust cellular heterogeneity resembling parental tumors was confirmed by several histological markers. By single-cell transcriptome analysis, the authors determined that both neoplastic and non-neoplastic cell populations (such as lymphocytes, macrophages, and microglia) retain parenteral molecular profiles after 2 weeks of culture. Orthotopic engraftment in an immunocompromised murine model was proven efficient, and aggressive infiltration was appreciated. The organoids were propagated by cutting them into 0.5 mm pieces. Cryopreservation protocols were also developed and optimized, and successful recovery was evident after thawing. Finally, the authors developed a biobank of organoids that allowed for testing different types of treatment *in vitro* (Jacob et al., 2020).

Hubert et al. (2016) reported the first effort to develop organoids for glioma research. The authors developed organoids from tumor tissue by modifying the original protocol described by Lancaster and Knoblich (2014). The group used finely minced patient tissue samples or their dissociated single-cell suspensions for this purpose. As initially described by Lancaster, they embedded the pearls of tissue in Matrigel and cultured them under shaking conditions to develop the organoids. Different from classical neurospheres, organoids grew until 3 to 4 mm after 2 months of culture. Similar to parenteral glioma tumors, the organoids developed a gradient of oxygen and stem cell density, delimiting a hypoxic core with quiescent glioma CSCs and a more oxygenated ring with proliferating CSCs. Xenograft tumors derived from different regions of the organoids (necrotic core and peripheral ring) showed different growth speed. Apart from showing a faster growth after xenotransplantation, cells in the necrotic core demonstrated higher radioresistance (Hubert et al., 2016). Worth to mention is that the culture methods for organotypic spheroids described by De Witt Hamer et al. (2008) were different from the organoid technology used by Hubert et al., which was based on the protocol published by Lancaster et al. (2013), Lancaster and Knoblich (2014).

The contemporary use of patient-derived organoids (PDOs) in general cancer research has led to some lessons: (1) Organoids can be generated from patient specimens; in general, either normal stem cells or CSCs can be used for this purpose (Gao et al., 2014; Bian et al., 2018; Ogawa et al., 2018); (2) organoid cultures can resemble interpatient variations and heterogenic intratumoral profile (Weeber et al., 2015; Tuveson and Clevers, 2019); (3) organoids represent a model to study the initiation, evolution, and drug response of the original brain tumor, allowing the identification of potentially targetable therapy (Hill et al., 2018; Lee et al., 2018; Tiriac et al., 2018). The U.S. Blue Ribbon Panel for the Cancer Moonshot has proposed to use these PDO as a screening tool for patient drug response (https://www.cancer.gov/research/key-initiatives/moonshot-cancer-initiative), and this effort has already shown initial evidence that PDO with specific genetic signature can help to identify a sizable number of patients with improved drug sensitivity.

However, even when PDO has been successful in representing patient therapeutic responses, there is still room for improvement, and as we have previously described, cocultured PDO or *in situ* glioma development in cerebral organoids has been engineered to include non-tumor TME cells such as immune cells (Dijkstra et al., 2018; Neal et al., 2018).

### Scaffolds

Scaffolds are 3D materials that provide support and structure to cell cultures; these biomaterials have microscale mechanical properties such as stiffness, porosity, interconnectivity, and structural integrity that can modulate cellular behavior (Mallick and Cox, 2013). For instance, biomaterial stiffness has been proven to affect stem cell differentiation through a number of pathways already described in the literature (Park et al., 2011; Palama et al., 2018; Xiao W. et al., 2019). In general, these properties as well as structural patterns, textures, and angulations can be controlled in an attempt to recapitulate ECM characteristics proper to the specific tissue of interest (Dijkstra et al., 2018). As the glioma TME possesses a distinct ECM composition with a high proportion of fibrillary collagens when compared to normal brain parenchyma (Huijbers et al., 2010; Lv et al., 2016), 3D glioma cultures using 3D collagen scaffolds have been studied with interest. Thus, a higher degree of dedifferentiation was found when compared to 2D cultures as well as a more similar morphology to *in situ* tumor GBM cells was also described. Furthermore, 3D cultures also showed greater resistance to alkylating agents with a high regulation of MGMT (Lv et al., 2016). Different scaffolds created with other relevant tumor ECM components such as HA as well as with synthetic materials have also been described (Erickson et al., 2018; Chaicharoenaudomrung et al., 2019; Xiao W. et al., 2019).

Conventional fabrication technologies used in scaffolds, such as the previously described, involve the use of physicochemical methods such as electrospinning (for polymers and biological materials), temperature-induced phase separation, and others (Lv et al., 2016; Erickson et al., 2018; Chaicharoenaudomrung et al., 2019; Xiao W. et al., 2019). In these cases, after the scaffold has been produced by the physicochemical procedures, the cells will be included in a posterior step as cell suspensions aiming to localize and home within the biocompatible scaffold.

Solid free-form (SFF) technologies, on the other hand, have recently positioned as one of the most relevant advances made in scaffolds fabrication. Among SFF technologies, 3D bioprinting has become a toolbox for a more tailored fabrication, allowing for better mimicking of the TME that now can include the tumor and non-tumor cells, together with biological ECM components such as macromolecules, and biomaterials.

#### 3D Bioprinting

**Figure 5:**
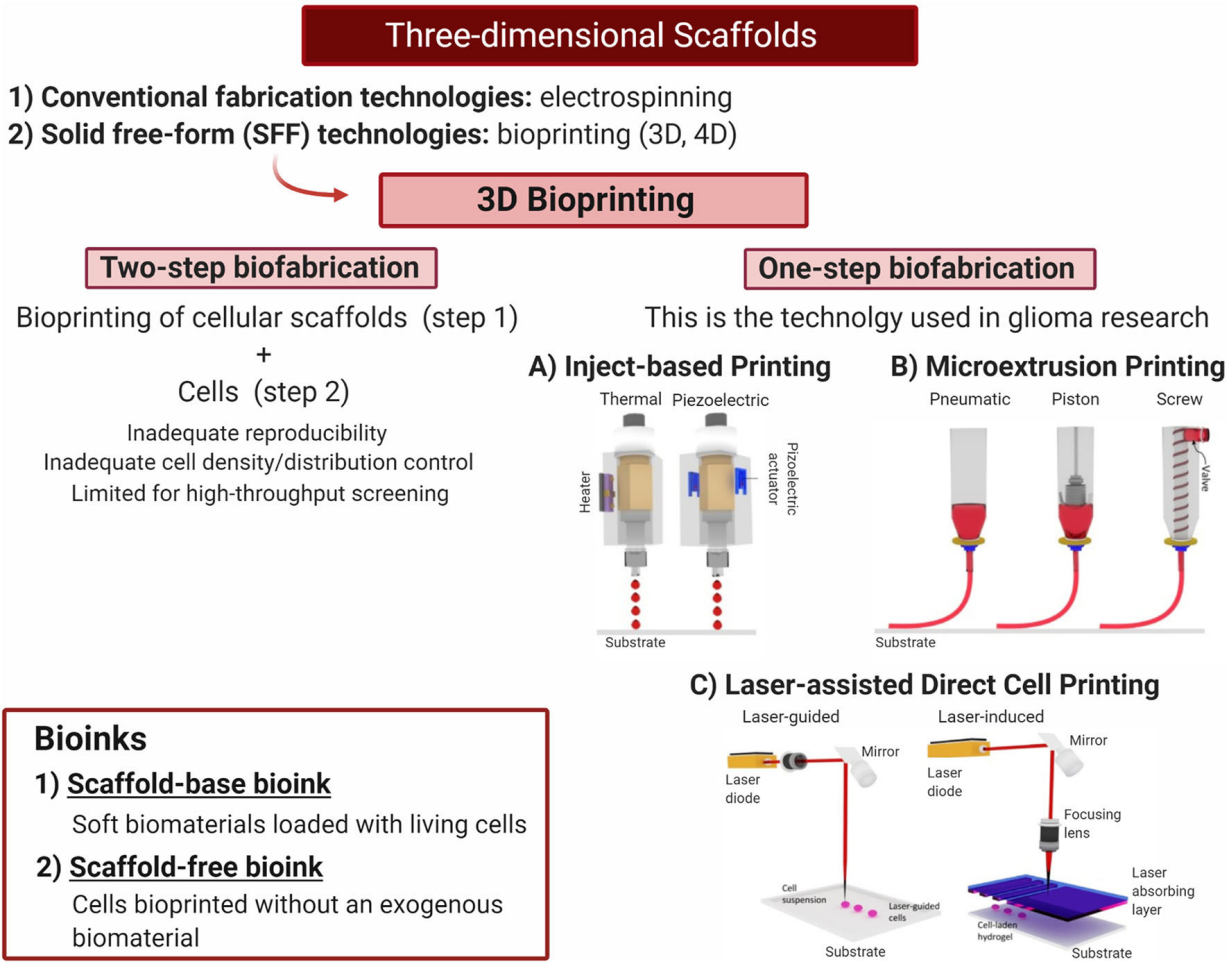
Three-dimensional scaffolds—one-step biofabrication techniques. Bioprinting is a highly promising tool to generate 3D microenvironments combining different biomaterial and cell lines to evaluate tumor growth and progression to generate new therapies. Current glioblastoma research has been developed using one-step fabrication techniques. **(A)** Thermal and piezoelectric inkjet printing. **(B)** Microextrussion printing. **(C)** Laser-assisted direct cell printing. Adapted with permission from Tasoglu and Demirci (2013). Created with BioRender.com.

3D bioprinting requires the use of bioinks to be deposited layer by layer, guided by a computer-aided design (Hospodiuk et al., 2017; Matai et al., 2020). There exist two types of bioinks: the first refers to soft biomaterials loaded with living cells (scaffold-base bioink), and the second refers to cells bioprinted without an exogenous biomaterial (scaffold-free bioink) (Dai et al., 2016). In the latter type of bioink, cells are grown up to small neotissues that are three-dimensionally distributed during the bioprinting process and will later fuse and mature to a more complex structure (Hospodiuk et al., 2017). Even when it is possible to create biosimilar acellular scaffolds using 3D bioprinting and later include a cellular component using the top-down method (two-step fabrication), this approach carries several limitations including inadequate reproducibility, cell density control, and spatial distribution control. Furthermore, the possibilities for high-throughput use are also limited (Tasoglu and Demirci, 2013). For this reason, one-step biofabrication techniques such as inkjet-based, microextrusion, and laser-assisted bioprinting are preferred (Asghar et al., 2015; Knowlton et al., 2015) (Figure 5). With these techniques, cells are located inside the 3D bioprinting while they are fabricated, thus reducing user input errors (Knowlton et al., 2015).

In glioma research, 3D bioprinting has been developed using one-step biofabrication techniques. Hermida et al. optimized a 3D model including alginates, ECM proteins such as collagen-1 and HA, plus U87-MG GBM cells, as well as stromal cells using the extrusion technique, and demonstrated to better represent therapeutic response when compared to 2D cultures (Hermida et al., 2019). Dai et al. (2016) described a similar approach, by using porous gelatin, alginate, and fibrinogen to simulate the ECM, mixed with U87-MG cells, the group bioprinted a 3D model of GBM where CSCs reach an 87% of survival and high rates of proliferation immediately after bioprinting. Furthermore, glioma cells were able to turn into a more differentiated neural cell population and the vascularized component of the model. In addition, higher chemoresistance was found in the 3D model when compared to 2D culture of glioma cells. (Dai et al., 2016) Wang et al. (2018a) used extrusion-based bioprinting technology to create a 3D glioma model to investigate the vascularization potential of patient-derived CSCs. Interestingly, a gel of gelatin, alginate, and fibrinogen was also used, and cell viability after impression was similar to the one reported by Dai et al. (2016) (86.27% ± 2.41%). Compared with cells grown in suspension, angiogenesis-related genes, *in vitro* vascularization potential, and stemness properties were more demarcated in the 3D model. Also, cellular ultrastructure in the 3D model showed more microvilli, mitochondria, and rough endoplasmic reticulum when compared to cells grown in suspension (Wang et al., 2018a).

In order to use 3D bioprinting to study the interaction between glioma CSCs and other non-tumor cells, Heinrich et al. (2019) developed a 3D-bioprinted mini-brain consisting of GBM cells and macrophages. The authors found that glioma cells communicate with macrophages, and trigger TAM polarization as described before in patients' tissue. Furthermore, macrophages would promote the EMT of GBM cells as evidenced by an increased expression of vimentin (Vim) and nestin (Nes), as well as a significant loss of E-cadherin (Cdh1). Consequently, higher glioma cell progression and invasiveness were noted in the mini-brains. When therapeutics target this intercellular communication, diminished tumor growth was recorded (Heinrich et al., 2019).

Similar approaches studying cell–cell and cell–ECM interactions in glioma CSC behavior and therapeutic response have also been reported (Dai et al., 2016; van Pel et al., 2018; Wang et al., 2018a,b, 2019; Haring et al., 2019; Heinrich et al., 2019; Mirani et al., 2019). Thus, current efforts in 3D printing for glioma research have focused on generating a better understanding of glioma biology, tumor angiogenesis, invasion, malignant transformation, drug susceptibility, and screening. These models are very promising in glioma research as they offer the possibility to manipulate and select specific factors to be studied according to any particular research question. The work of Heinrich et al. (2019) shows that it is possible to include more than one cell type within the gels, which allow for studying cell–cell interactions. We envision that this technology will help to dissect and understand in much more detail the very complex network of communications between different cell types. Additionally, as the manipulation of the physical properties of the gels is feasible, this will allow inquiring how the physical properties of the ECM affect glioma biology and test processes such as mechanotransduction. Overall, the development and refinement of this technology are highly relevant in the understanding of glioma CSC biology.

## Advances in Glioma Research Using 3D Models

The use of 3D biomaterials used to simulate ECM mechanical properties and cell–ECM interactions has led to a deeper understanding of the mechanobiology underlying tumor malignancy, cancer cell migrations, and resistance to therapies. Furthermore, the previously described developments have allowed generating even more complex technologies to better study the relationship between not only the TME but also the interaction with the whole human body.

### Organ-On-a-Chip

Organ-on-a-chip is a new technology that combines tissue engineering technologies with microfluidics to develop artificial systems that can recreate organ functions, organ interactions, and human physiology (Zhang et al., 2018). Yi et al. (2019) showed that an organ-on-a-chip GBM model that matched the clinical outcome after concurrent chemoradiation with temozolomide exhibited patient-specific sensitivity against specific drugs combinations. The interaction within the perivascular niche has also been studied using this technology, suggesting that glioma CSCs located around the vasculature and presenting with the lowest motility are most probably of the proneural subtype, and those with the highest invasiveness are most probably classified in the mesenchymal subtype; which further supports the role of the tumor niche on intratumor heterogeneity and consequent treatment response (Xiao Y. et al., 2019). Studies regarding GBM response to magnetic hyperthermia have been also carried out in a similar way (Mamani et al., 2020).

### Four-Dimensional Bioprinting

Four-dimensional (4D) bioprinting is emerging as the next generation for biofabrication technology. Different from 3D bioprinting, which is static, 4D bioprintings introduce the use of stimuli-responsive biomaterials that can be modified in a time-depended manner (fourth dimension) in an attempt to mimic the physiological activities proper of any living microenvironment (Ashammakhi et al., 2018; Truong et al., 2019; Yang et al., 2019). 4D bioprinting has been used for drug screening, drug delivery, and vascularization models; therefore, this technology could help in the comprehension of glioma progression and therapy (Gao et al., 2016; Ruskowitz and DeForest, 2018).

## Conclusions and Future Perspectives

The use of preclinical 3D models represents an opportunity to better understand glioma biology, as well as to perform high-throughput screening able to accelerate the selection of the most effective and personalized therapy for individual patients. To maximize the advantages of these models, they should rigorously represent most factors characterizing the TME, having in mind not only its cellular and non-cellular components but also the biomechanics underlying their interactions. Thus, it will be important to differentiate the characteristics we must represent in order to simulate the different tumor regions such as the core, the external layers, and even the surrounding healthy tissue that gliomas CSCs will inevitably infiltrate. Modeling each of these different regions will be fundamental to better study the heterogeneous CSC phenotypes, behaviors, and treatment responses; which in turn will be crucial to find a clinically relevant alternative for glioma patients.

## Author Contributions

HR-G and KA-E reviewed the literature and wrote the first draft of the manuscript. HR-G created the figures. PS, AQ-H and DT critically revised the manuscript. HR-G, KA-E, AQ-H and DT worked on the study conception and design. All authors analyzed the data, drafted the manuscript, and read and approved the final version of this work.

## Conflict of Interest

The authors declare that the research was conducted in the absence of any commercial or financial relationships that could be construed as a potential conflict of interest.
